# Supplementary material for: Tradeoff between lag time and growth rate drives the plasmid acquisition cost
Source: Nat Commun. 2023 Apr 24;14:2343. doi: 10.1038/s41467-023-38022-6 (PMC10126158; doi:10.1038/s41467-023-38022-6)
Supplement: Supplementary file 1 — Supplementary information [file 41467_2023_38022_MOESM1_ESM.pdf]

Supplementary Information For

Tradeoff between lag time and growth rate drives plasmid acquisition costs

Mehrose Ahmad[1], Hannah Prensky[1], Jacqueline Balestrieri[1], Shahd ElNaggar[1], Angela Gomez-Simmonds[2], Anne-Catrin Uhlemann[2], Beth Traxler[3], Abhyudai Singh[4], Allison J. Lopatkin[1,5,6-8,*]

[1]Department of Biology, Barnard College; New York, NY 10027; USA
[2]Department of Medicine, Division of Infectious Diseases, Columbia University Medical Center, New York, NY 10032
[3]Department Microbiology, University of Washington; Seattle, WA 98195; USA
[4]Department of Electrical and Computer Engineering, University of Delaware; Newark, DE 19717; USA
[5]Department Ecology, Evolution, and Environmental Biology, Columbia University; New York, NY 10027; USA
[6]Data Science Institute, Columbia University; New York, NY 10027; USA
[7]Department of Chemical Engineering, University of Rochester; Rochester, NY 14627; USA
[8]Department of Microbiology and Immunology, University of Rochester Medical Center; Rochester, NY 14627; USA
[*]Corresponding author: allison.lopatkin@rochester.edu

This file includes:

Model development
Supplementary Figures 1-9
Supplementary Tables 1-4

Model development

Overview of logistic model for constrained growth/lag relationship

To model the ecological implications of the observed tradeoff between lag time and growth rate, we developed a consistent method to constrain growth rates by observed lag times (i.e., the presence of a tradeoff), or lack thereof, for use in all subsequent simulations. Specifically, individual clonal growth curves were fit using the modified logistic growth equation[1]:

(S1) $$N_i = \frac{A_i}{\left[1+e^{\frac{4\mu_{m_i}}{A_i}(\lambda_i - t)+2}\right]}$$

Here, μ_{m_i} and λ_i are taken to be the maximum growth rate and lag time for each ith individual clone, respectively, A_i is the maximum density per clone, and N_i is the log-transformed cell density, as described in Methods. Linear regressions were then used to correlate fitted lag times, λ_i, to their corresponding growth rates, μ_{m_i}, yielding the fits shown in Fig. 3a (black dotted lines). The linear regressions for each plasmid resulted in a slope m_p defined as: $m_p = \frac{(\lambda_i - \lambda_P)}{(\mu_{m_i} - \mu_P)}$, where λ_P and μ_P represent the mean lag time and growth rate of a particular plasmid-carrying transconjugant. Thus, a statistically significant slope greater than zero was indicative of a tradeoff between lag time and growth rate; a slope less than or equal to zero and implied a lack of tradeoff. Experimental data revealed that the tradeoff slope was a linear function of the acquisition cost (Fig. 4a) and lag time (Supplementary Fig. 6a) across all plasmids investigated. Therefore, we used this continuous relationship (Eq. S2) to determine the slope of any plasmid tradeoff:

(S2) $$m_p = 1.31 * \lambda_p - 12.39.$$

To simulate intrapopulation heterogeneity (i.e., to simulate variability amongst individual clones belonging to a single genetically identical population), where applicable, simulated lag times were randomly and independently drawn from a lognormal distribution parametrized by the mean and standard deviation of experimentally collected lag times. Lognormal distributions were chosen based on the literature consensus of this distribution type best fitting lag times, which was consistent with our data[2,3]; normal distributions produced qualitatively similar results. Growth rates were then calculated using this *a priori* parameterized slope, relative to the mean lag time and growth rate of the plasmid (constrained, below), or randomly drawn from a normal distribution with the mean and standard deviation equal to that from the data (unconstrained):

(S3) $$\mu_{m_i} = \frac{1}{m_p}(\lambda_i - \lambda_P) + \mu_P(1 + \delta_P N(0,1))$$ (constrained)

(S4) $$\mu_{m_i} = \mu_P(1 + \delta_P N(0,1))$$ (unconstrained)

where δ_P represents the coefficient of variation associated with μ_P and $N(0,1)$ corresponds to the standard normal distribution. As detailed below, this linear regression framework was subsequently used to implement the presence/absence of a tradeoff in various simulation scenarios.

Bootstrapping method to predict time-to-threshold from lag/growth tradeoff (Fig. 3b)

To verify the presence and criticality of the lag/growth tradeoff in predicting heterogeneous transconjugant dynamics, we utilized a bootstrapping approach to capture our acquisition cost data. Specifically, we simulated various *de novo* plasmid-bearing populations, defined by unique $\{\mu_P, \lambda_P\}$ values. Here, we aimed to ascertain the likelihood that observed lag/growth tradeoffs (i.e., Fig. 3a) arose randomly or through numerical artifact, or rather if they are essential to accurately predict observed times-to-threshold. Since experimentally, we quantify acquisition costs by tracking individual clones grown independently on an agar plate, we used the logistic model above (Eq. S1) to simulate the growth of all clones in the population without direct competition. We generated 300 independent clones either with or without a lag/growth tradeoff (Eq. S3-4), and simulated their growth for 48 hours using Eq. S1. The predicted time-to-threshold (TTT_P) could then be extracted from each growth curve using the same threshold as in experiments (0.8), and its dependence on growth rate was calculated based on a linear regression across all clones. This process was repeated 1000 times to yield a distribution of predicted TTT/growth rate regressions. In the constrained case, this distribution represents the pooled effects of the lag/growth tradeoff as well as intrapopulation variability; in the unconstrained counterpart, only intrapopulation variability is reflected. As described in the main text, we found that for all *de novo* populations with experimentally verified $m_p > 0$, an unconstrained model lacking this tradeoff was unable to accurately predict the observed relationship between TTT and growth rate. Statistically, in the absence of tradeoff, the observed slope fell outside the 95% confidence interval of the predicted slope distribution. Conversely, simulating the relevant tradeoff was sufficient to capture the experimental slope value (observed value within 95% confidence interval of predicted distribution) (Fig. 3b). In other words, the tradeoff between lag time and growth rate was necessary to capture the experimentally observed TTT; variability within clonal populations was not sufficient to explain this relationship. Intuitively, in the absence of a tradeoff, clones that grow faster reach TTT sooner, as seen in the adapted case. However, this negative correlation is not observed in *de novo* cells, which would only be predicted with a growth/lag tradeoff.

ODE model development

Our data clearly shows that acquisition costs are primarily driven by lag time, rather than growth rate. Thus, to simulate the population dynamics of *de novo* and adapted transconjugants, and the implications of acquisition cost, lag time, and growth rate relationships, we used a simplified set of ordinary differential equations (ODEs) that describe clonal growth and competition:

(S5) $$\frac{dn_i}{dt} = n_i\,\mu_i\,(1 - \frac{\sum_{i=1}^{J} n_i}{N_m})$$

where $\mu_i = 0$ if $t < \lambda_i$, and $\mu_i = \mu_{m_i}$ if $t \geq \lambda_i$. Here, N_m represents the shared carrying capacity, μ_i is the maximum growth rate for each ith clone, and the initial population consists of J total clones. All model

parameters can be found in Supplementary Table 4. Generally, *de novo* and adapted simulations differ primarily in the lag times, while their growth rates are the same (Supplementary Fig. 3c). In all cases, simulations were run for 48 hours to ensure accurate calculations of growth rates from the resulting curves. All presented distributions and statistics of simulation-generated lag times, growth rates, and population fractions were calculated based on multiple independent iterations of the relevant simulation initiated from identical conditions. In a given iteration, each clone $n_{1..J}$ was assigned a lag time λ_i drawn from the corresponding lognormal distribution with a mean and standard deviation as listed in Supplementary Table 4; corresponding growth rates μ_i were calculated as described above, depending on whether a tradeoff constraint was implemented.

Simulating *de novo* and adapted transconjugant variability

Consistent with experimental results, simulated *de novo* and adapted populations were differentiated by their $\{\mu_P, \lambda_P\}$ values, as well as by the slope governing any tradeoff between these two parameters. Furthermore, for all *de novo* simulations, we simulated the background parent density remaining after conjugation (See Methods), since depending on the number of transconjugants after the 1 hour conjugation period, dilutions were sufficiently low such that these cells contributed to carrying capacity effects experimentally, even if they are unable to grow. Specifically, we assume that all parents were at least 1000X higher in initial density than corresponding *de novo* cells, but had a growth rate and lag time equal to 0, as the presence of dual antibiotic prevented their growth. For example, in a simulation with 100 unique *de novo* transconjugants (i.e., *J*=100), 100 independent populations (i.e., equations) were each initiated from independent singles clone with uniquely defined parameters and an initial density of 100 cells. Therefore, the parent population was initiated with a density of 1000*100= 1e5 cells. This is consistent with our experimental data, where low initial dilutions with high parental densities inhibited any transconjugant growth; in contrast, at sufficiently high dilution, residual parental density was minimal, allowing transconjugants to grow (Supplementary Fig. 6b). In all cases, 50 independent iterations of each simulation were run to obtain an estimate of variation in the dynamics as driven by the distributions; the averages and standard deviations are reported.

Tradeoff results in a density-dependent growth advantage (Fig. 4b-c)

To simulate intrapopulation competition (i.e., Fig. 4b-c), competitions of varying *J* number of initial populations (i.e., clones) were used, from ~30 to ~300,000, where each population was treated as a unique clone at time=0 with distinct parameters (x-axis in figure). Thus, heterogeneous populations of increasing size were simulated, and resultant growth rate captured. To capture the growth rate equivalent to that observed experimentally, the parameters setting the growth rates for each clone (E.g., Eq. S3-4) are not sufficient, as these values do not scale with the number of populations, *J*; that is, an observed growth rate may potentially be limited due to total density (i.e., carrying capacity effects) even if the underlying parameterized growth

rates of individual cells is high. Thus, for each iteration, the clone that reached the maximum density after 48 hours was selected (i.e., the "winner"), and an *observed growth rate and lag time for that clone* were calculated using the logistic method, equivalent to our experimental approach. We note that the *average* growth rates used to generate initial variability were chosen to match experimental data; however, the observed *de novo* growth rate is confounded by variability in individual cells' time since conjugation; at any given time, even a phenotypically homogeneous population will contain cells that have yet to begin growing, thereby depressing the observed growth rate. The mean and standard deviation of winner growth rates from 50 iterations are presented; changing the underlying mean growth rate only shifts the curves up or down, but does not alter the qualitative trends (Supplementary Fig. 6c). Specifically, growth rates for both R100-1 populations are set to 0.4, whereas *de novo* RP4 was set to 0.3, and adapted RP4 to 0.5. Finally, the variance in these simulations was chosen to be low to capture the clonal heterogeneity while remaining biologically feasible (0.002).

<u>Intermediate acquisition costs confer a competitive advantage (Fig. 4d-f)</u>

To simulate generic inter-plasmid competition (i.e., Fig. 4d-f), we used the same set of equations to simulate two sub-populations, each corresponding to cells carrying one of two plasmids. Parameters for clones in each sub-population were drawn from independent distributions using the same approach as described above, each centered around the mean for that particular plasmid. To investigate ecological outcomes (i.e., final population fractions) across all possible plasmid cost combinations (i.e., Fig. 4d), we simulated pair-wise competitions between two populations: a reference plasmid, and a second whose cost was varied (Fig. 4d, x-axis) by increasing lag times between 8-18h. The reference population's parameters were fixed at the median lag time to represent a relatively medium cost plasmid (red vertical lines in Fig. 4d). In this way, pair-wise competitions captured the population dynamics of a medium cost plasmid against a plasmid with all other possible acquisition costs. To simulate competitions with and without the tradeoff (i.e., Fig. 4d right and left respectively), growth rates were chosen using the constrained or unconstrained methods as defined above (Eq. S3-4). In all cases, 50 iterations of each simulation were averaged; the mean and standard deviation population fraction of the medium cost reference plasmid are reported.

To simulate specific experimental competitions, as opposed to the range of lag times described above, we used specific parameter estimates based on the corresponding plasmids. Specifically, we first simulated competition between adapted sub-populations; their initial densities were scaled to ensure that they reached equivalent population fractions after 48 hours of growth (Supplementary Fig. 8), in line with experimental observation. These initial conditions were then used as the starting densities for *de novo* competitions, with the lag times and slopes modified accordingly (Supplementary Table 4). Thus, any growth (dis)advantages enjoyed/suffered by *de novo* populations were easily distinguishable. We note that for these simulations, we used a higher variance to better capture inter-population dynamics (0.1, Fig. 4e-f); however, this did not

impact the overall results, and the same variance as in all Figure 4 simulations can be used (Supplementary Fig. 9).

Figures

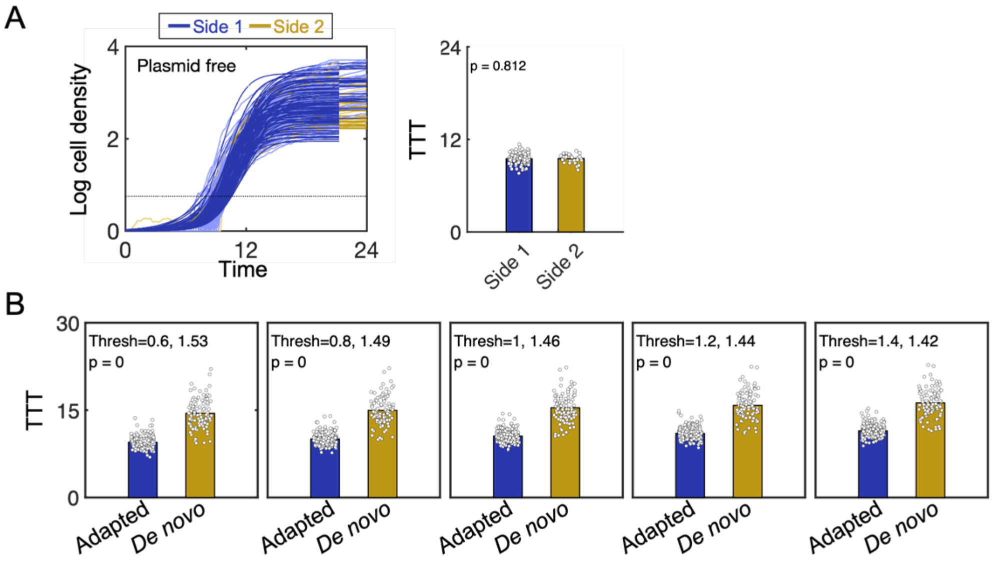

Supplementary Figure 1: Acquisition cost quantification controls.

A. **Control for Agar Plating:** To assess if individual sides of a single agar plate impacted the plasmid acquisition cost, we artificially split plasmid-free recipient RB933, and plated each population on separate sides of a rifampicin-containing agar plate. *Left:* Log cell density in pixels (y-axis) of each population (yellow or purple) over time in hours. The black horizontal line indicates the time-to-threshold (TTT) in hours. *Right:* The TTT in hours (y-axis) of both populations (yellow or purple). Each marker corresponds to the TTT of an individual colony (n=104 for Side 1; n=32 for Side 2). Bar height represents the mean TTT of three biological replicates, and the reported p value is calculated using a two-sided t-test.

B. **Control for Chosen Threshold Density:** The acquisition cost of intermediate-cost plasmid RP4 was assessed using different TTT values of 0.6, 0.8, 1, 1.2, and 1.4 (shown from left to right). Values listed next to the thresholds represent RP4's plasmid acquisition cost at that respective threshold. Each marker corresponds to the TTT of an individual colony. Bar height represents the mean TTT of at least three biological replicates. Colony numbers for both adapted and *de novo* populations are the same as those reported in Fig. 2a (n) and listed in Supplementary Table 1c. The reported p values are calculated using a Bonferroni-corrected two-sided t-test.

In all cases, TTT is defined as the time in hours it takes a bacterial population to reach a 'threshold' density in exponential phase. All Source Data are provided as a Source data file.

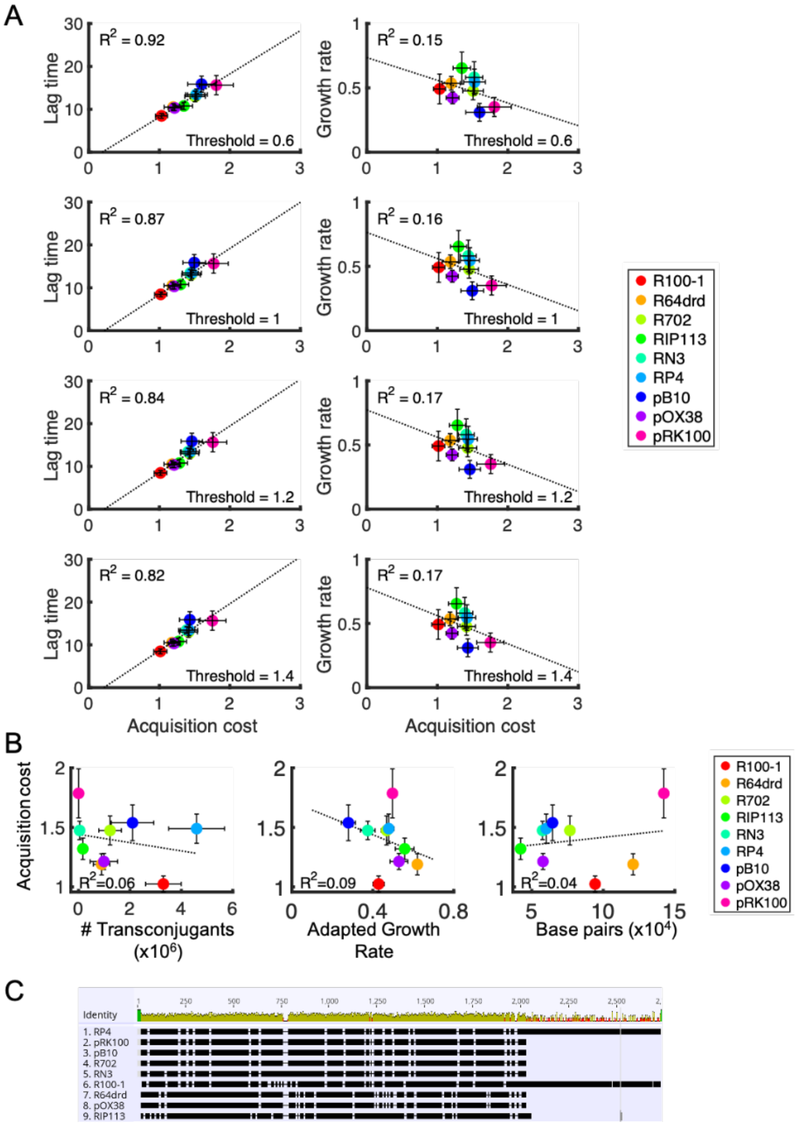

Supplementary Figure 2: Generality for tetracycline-specific phenotypes

A. **Acquisition cost quantification is independent of threshold density:** Growth rate (hr^{-1}) and lag time (hr) trends are maintained when a threshold density of either 0.6, 0.8 (main figures), 1.0, 1.2, or 1.4, is used to determine the time-to-threshold for all nine plasmids measured under tetracycline selection.

B. **Acquisition cost does not correlate to general plasmid features:** Relationships between plasmid acquisition cost and various plasmid-specific traits, including transconjugant number, adapted growth rate, or plasmid length, are shown. Linear regressions in all cases are statistically insignificant ($p > 0.5$).

C. **Multi-sequence alignment of all Tet operons.** Each Tet operon was extracted as described in the Methods. Alignments were visualized in Geneious prime.

For B-C, each individual marker represents a distinct plasmid (Red: R100-1; Orange: R64drd; Chartreuse: R702; Green: RIP113; Mint: RN3; Light blue: RP4; Dark Blue: pB10; Purple: pOX38; Pink: pRK100). Black lines represent the linear regression line of best fit, the R^2 value is reported, and error bars represent standard deviations. For 2a-b, markers indicate the means, and error bars the standard deviations, for all n *de novo* colonies; n in each case is pooled across at least three independent biological replicates, and all sample sizes can be found in Supplementary Table 1c.

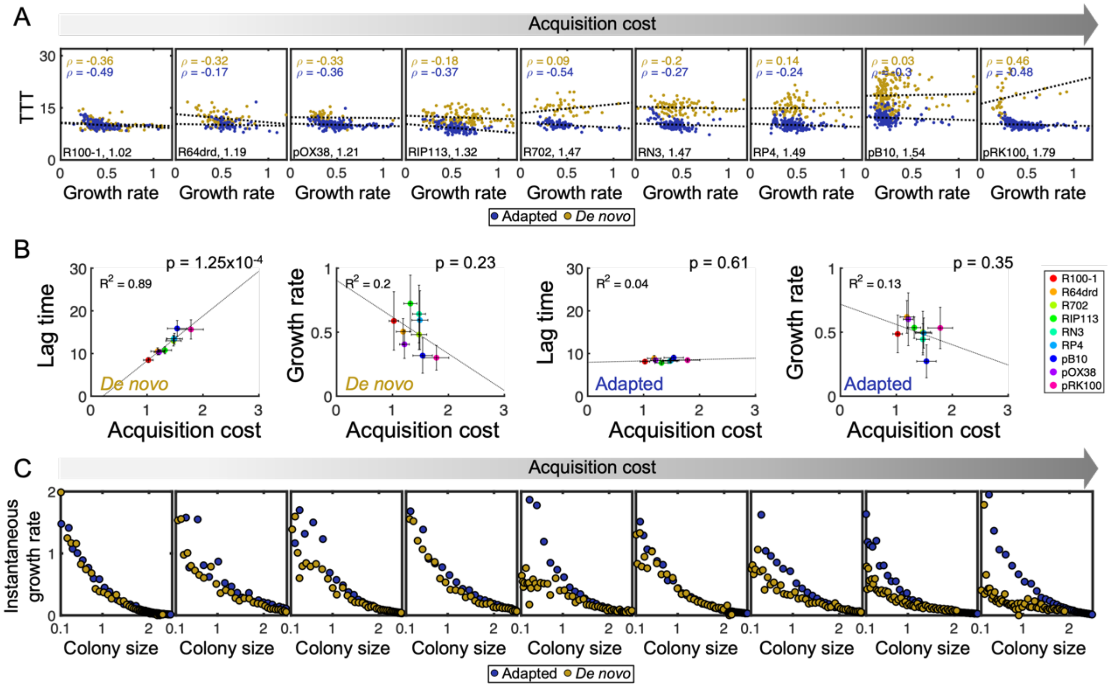

Supplementary Figure 3: Growth rate validation.

A. Individual plasmid trends maintained using alternative growth rate quantification. Data from Fig. 2a, middle panel, is analyzed using the manual growth rate method. In all cases, dashed lines represent the linear regression of best fit, and Pearson correlation coefficient (ρ) values are shown. Colony numbers (n) for both adapted (purple) and *de novo* (yellow) populations are the same as those reported in Fig. 2a. Plasmid acquisition costs are reported from the average of all n colonies across at least 3 biological replicates as reported in Supplementary Table 1c.

B. Acquisition cost trends maintained using alternative growth rate quantification. Same trends as in Fig. 2b were maintained using the manual growth fitting method. P-values are reported for the slope of the linear regression. Each individual marker represents the mean lag time (or growth rate) and acquisition cost for all n colonies of a particular plasmid (Red: R100-1; Orange: R64drd; Chartreuse: R702; Green: RIP113; Mint: RN3; Light blue: RP4; Dark Blue: pB10; Purple: pOX38; Pink: pRK100). In all cases, colonies are pooled across at least three independent biological replicates (see Supplementary Table 1c for all sample sizes), black lines represent the linear regression line of best fit, and the R^2 and p values are reported.

C. Instantaneous growth rate. Adapted (purple) and *de novo* (yellow) colony sizes were determined by averaging the pixel number for all colony replicates within a single plasmid, and the instantaneous growth rate (hours^{-1}) was calculated by: $\log(x(t)/x(t-1))/0.25$, where x is the number of pixels at time t. Colony numbers are the same as those reported in Fig. 2b.

All Source Data are provided as a Source data file.

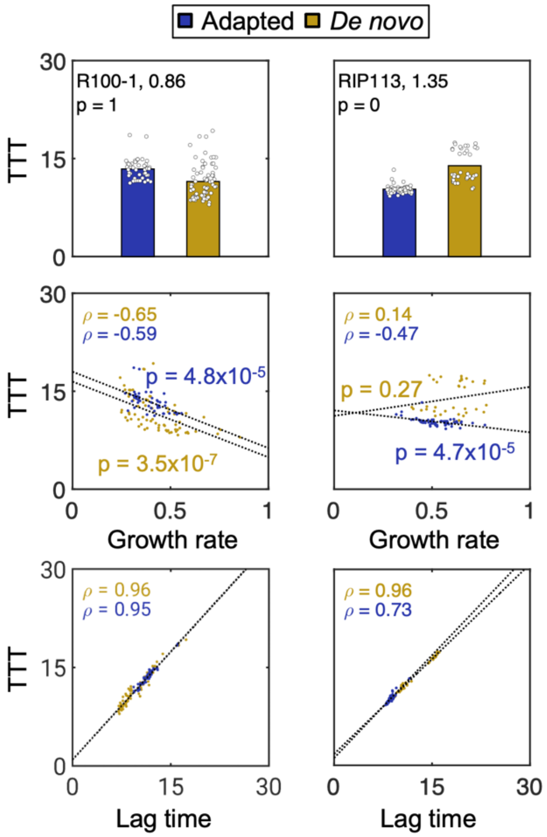

Supplementary Figure 4: Acquisition cost results are independent of the recipient selection agent. Acquisition costs of plasmids R100-1 and RIP113 using the recipient strain RB933 were measured under tetracycline/kanamycin selection instead of tetracycline/rifampicin. Left: for the R100-1 plasmid, the TTT in hours (y-axis) of *de novo* (n=71, yellow) and adapted transconjugants (n=46, purple) plotted as a function of either growth rate in hours^{-1}or lag time in hours (x-axes). Right: for the RIP113 plasmid, the TTT in hours (y-axis) of *de novo* (n=35, yellow) and adapted transconjugants (n=62, purple) plotted as a function of either growth rate in hours^{-1}or lag time in hours (x-axes). In all cases, each marker corresponds to the TTT of an individual colony; bar height represents the mean TTT of at least two biological replicates. Black lines represent the linear regression line of best fit for each population; acquisition costs, Pearson correlation coefficient (ρ) values, and p values are reported for the slope of the regression line. All Source Data are provided as a Source data file.

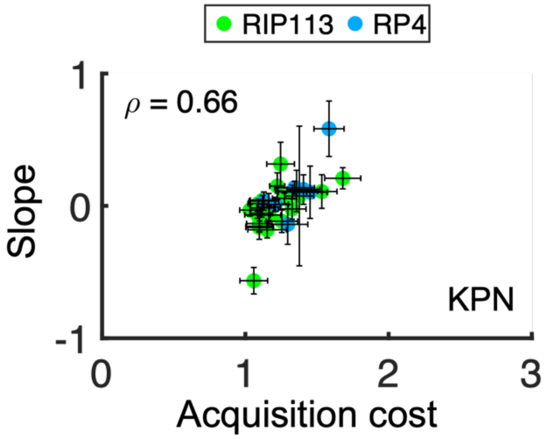

Supplementary Figure 5: Growth/lag tradeoff in *Klebsiella pneumonia.* 24 unique *Klebsiella pneumonia* strains conjugated with intermediate-cost plasmid RIP113 (green), and 7 unique *Klebsiella pneumonia* strains conjugated with intermediate-cost plasmid RP4 (light blue), were pooled. The slope of the regression line between growth rate and lag time was determined for each strain. This slope was plotted as a function of acquisition cost. The Pearson correlation coefficient (r) value is reported (r = 0.66). For each data point, error bars represent standard deviations and acquisition costs represent means of n colonies across two independent biological replicates as listed in Supplementary Table 1c. All raw data are provided as a Source data file.

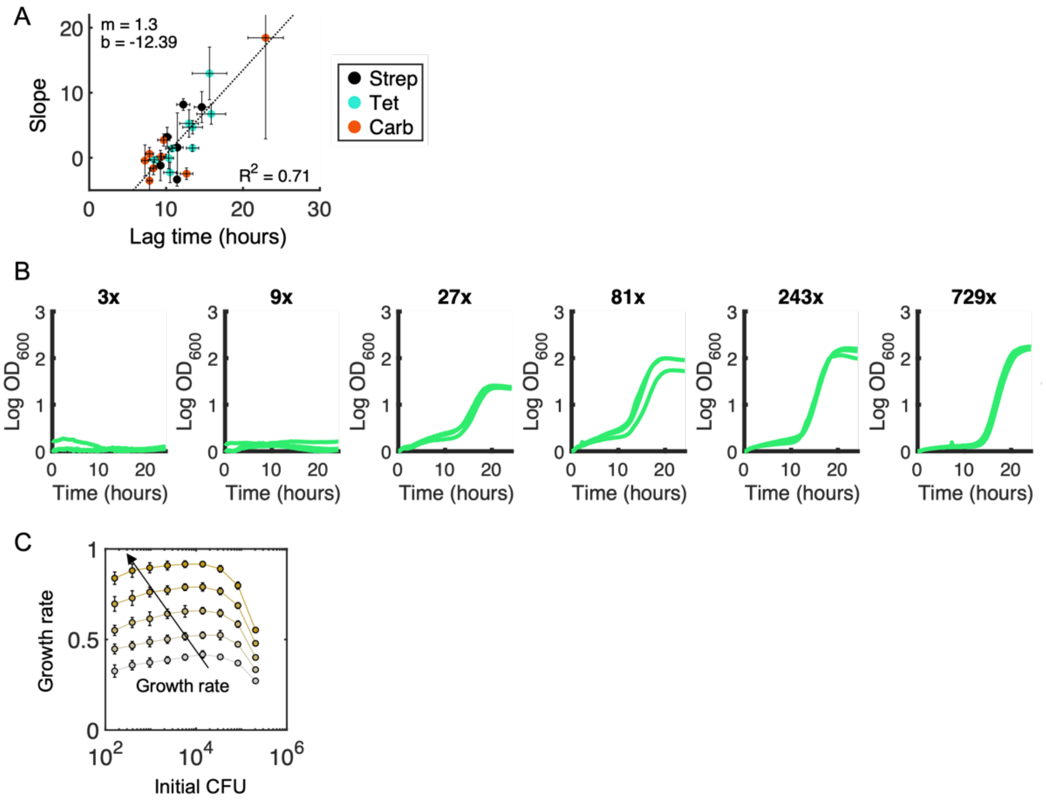

Supplementary Figure 6: Model validation

A. Lag time and slope linear dependence. *De novo* lag times (hours) are linearly correlated with the strength of lag time/growth rate tradeoff (i.e., Fig. 3a) across all antibiotics (streptomycin in black, tetracycline in blue, and carbenicillin in orange). Data is the same as in Fig. 4a. Markers represent the mean, and horizontal error bars represent the standard deviation, of lag times for the indicated n number of *de novo* colonies listed in Supplementary Table 1c; vertical error bars represent the 95% confidence interval corresponding to the slope estimates from Fig. 3a. R^2 and slope (m) and y-intercept (b) values of the linear regression line are reported.

B. Parent populations impact carrying capacity. pB10 conjugation mixtures, consisting of donors, recipients, and *de novo* pB10 transconjugants, are diluted in steps of 3-fold (increasing from left to right) into a dual-antibiotic mixture of tetracycline and rifampicin. Cells are incubated over time in a plate reader. Only when the background population is sufficiently diluted is there room for *de novo* transconjugants to grow. Given day to day variation in transconjugant numbers, the primary source of variability arises in the diluted wells at lower cells density; therefore, experiments were done in technical triplicates from a single biological replicate.

C. Growth rate does not change qualitative biphasic trends. Intrapopulation heterogeneity results for the RP4 plasmid (Fig. 4b) are independent of the *de novo* growth rate ($hours^{-1}$). Marker color from gray to dark yellow indicate growth rates from 0.2 to 0.6 (main figure is 0.3), and black arrow indicates direction of increasing growth rate. Error bars represent standard deviations determined from n=50 iterations.

All Source Data are provided as a Source data file.

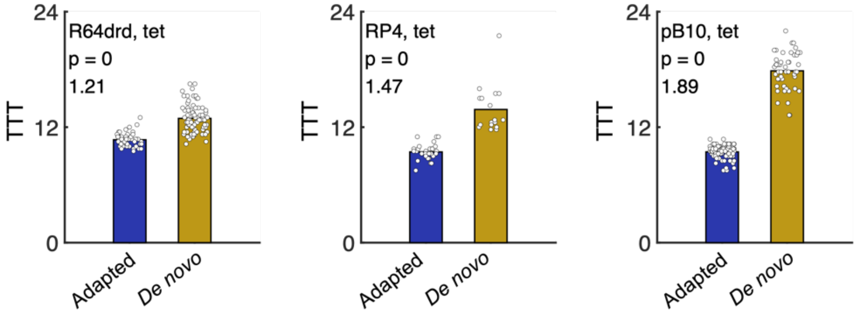

Supplementary Figure 7: Plasmid acquisition cost in BW25113-Rif recipient. Acquisition costs of plasmids in recipient strain BW25113-rif were quantified and their p values from Bonferroni-corrected two-sided t-tests are reported; plasmids are organized in order of increasing acquisition cost (left to right). Individual markers represent individual TTTs (in hours) for unique adapted (purple, n=50, 51, 88 left to right) or *de novo* (yellow, n=77, 17, 51 left to right) colonies. Acquisition cost results are qualitatively consistent with those in RB933 recipient strain. All Source Data are provided as a Source data file.

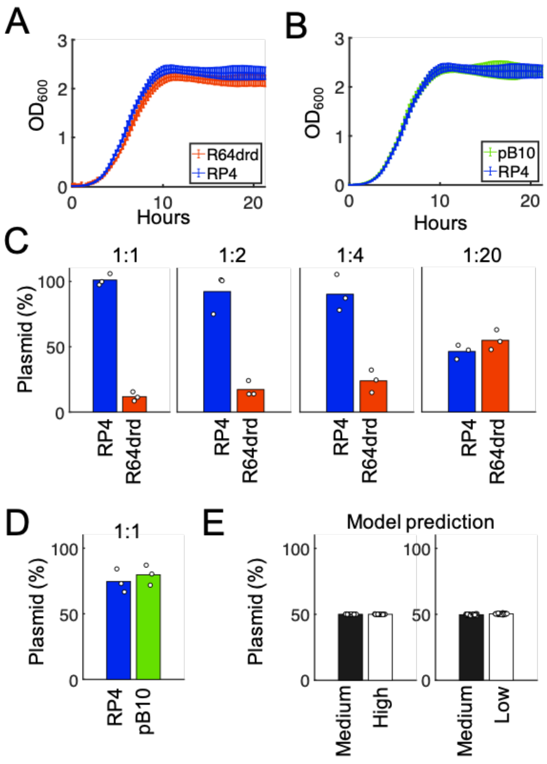

Supplementary Figure 8: Inter-plasmid competition calibration.

A. **Adapted RP4 and R64drd time courses.** Representative time courses are shown for low-cost plasmid R64drd (red) and intermediate-cost plasmid RP4 (blue) adapted transconjugants.

B. **Adapted pB10 and RP4 time courses.** Representative time courses are shown for high-cost plasmid pB10 (green) and intermediate-cost plasmid RP4 (blue) adapted transconjugants.

For A-B, the y-axis is OD_{600} and x-axis is time in hours. Error bars represent standard deviations of averages of three technical replicates, and n=3 biological replicates were measured. All plasmids are in recipient strain BW25113-rif. All Source Data are provided as a Source data file.

C. **Adapted RP4 and R64drd at varying initial ratios.** Adapted transconjugants of intermediate-cost plasmid RP4 (blue) and low-cost plasmid R64drd (red) were competed at the following ratios, with each ratio representing the relative amount of cells containing RP4 and R64drd, respectively: 1:1, 1:2, 1:4, and 1:20 (left to right).

D. **Adapted RP4 and pB10 at varying initial ratios.** Adapted transconjugants of RP4 (blue) and pB10 (green) were competed in a 1:1 ratio representing RP4 and pB10, respectively.

For both C-D, each plasmid's population fraction percentages were calculated by dividing the CFU from each unique drug plate (i.e., carbenicillin/rifampicin for RP4 and streptomycin/rifampicin for R64drd and pB10) by the total CFU from tetracycline/rifampicin, multiplied by 100. Each competition was conducted with n=3 biological replicates; each marker represents a unique biological replicate with each bar height representing the average of all biological replicates. All plasmids are in recipient strain BW25113-rif. All Source Data are provided as a Source data file.

E. **Model validation of adapted competitions.** Modeling adapted competitions using appropriate ratios and confirmed corresponding conditions achieve equal final population fraction. Bar height represents averages, error bars represent standard deviations, of 50 iterations.

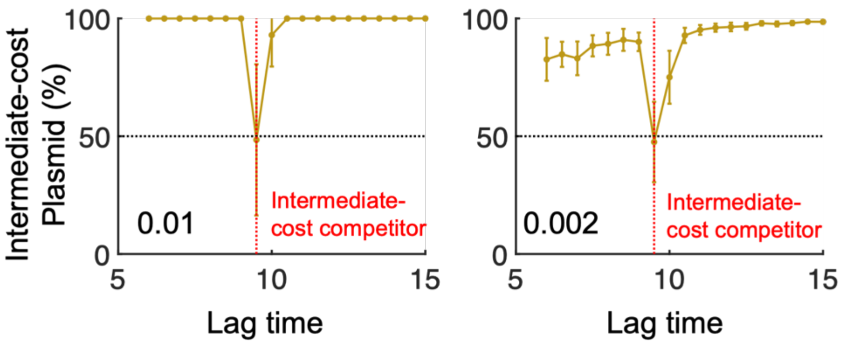

Supplementary Figure 9: Variance does not significantly alter competition outcomes. To determine the robustness of results from Fig. 4d, competition simulations were run at different variance levels. Compared to the main data (variance=0.1), decreasing variance to 0.01 and 0.002 does not qualitatively impact the main results; in the presence of a lag/growth tradeoff, intermediate-cost plasmids can outcompete plasmids of either lower or higher cost. Data points represent the mean of 50 iterations, whereas error bars indicate standard deviations.

Tables

Supplementary Table 1: Strains, plasmids, and antibiotic conditions used in this study

A. List of recipients:

Name	Selection[1]	Strain Genotype	Source
RB933	kanamycin (only in Supplementary Fig. 4) rifampicin	*E. coli* (ΔlacIZYA::scar galK::catYFP ΔgatZ::FRT-aph-FRT, rpoBH526Y)	Generous gift from I. Gordo (Cardoso et al, 2020)[4]
BW25113-rif	rifampicin	*E. coli* BW25113 (F^-, Δ*(araD-araB)567,* Δ*lacZ4787(::*rrnB-3*),* λ−*, rph-1,* Δ*(rhaD-rhaB)568, hsdR514, rpoB*^{M}*)*	This paper
KPN	rifampicin	0029, 0043, 0047, 0049, 0054, 0064, 0087, 0106, 0112, 0127, 0159, 0165, 0169, 0193, 0200, 0201, 0212, 0224, 0229, 0243, 0267, 0284, 0306, 0337	Generous gift from A. Simmonds and A. C. Uhlemman (Gomez-Simmonds et al, 2015)[5]

[1]Working concentrations of drugs are as follows: kanamycin (50 μG/mL), rifampicin (50 μG/mL),

B. List of donors:

**Plasmid**	**Inc**	**Length (bp)**	**Average CFU in cells/mL (log_{10})[1]**	**Standard Deviation in cells/mL (log_{10}) [1]**	**Resistance[2]**	**Donor strain**	**Source**
R100-1	F	94282	6.49	0.19	chloramphenicol spectinomycin streptomycin **tetracycline**	*E. coli* DH5*a*	Generous gift from D. Mazel (Baharoglu et al, 2010)[6]
R16a	A/C	173094	3.71	0.45	**carbenicillin** kanamycin	*E. coli* MG1655 *Δara*	Generous gift from F. Dionisio and J. Alves Gama (Gama et al, 2020 and Prensky et al, 2021)[7,8]
R1drd	F	97735	5.07	1.46	**carbenicillin** chloramphenicol kanamycin spectinomycin streptomycin	*E. coli* MG1655 *Δara*	Generous gift from F. Dionisio and J. Alves Gama (Gama et al, 2020 and Prensky et al, 2021)[7,8]
R57b	A/C	170470	3.54	0.04	**carbenicillin** chloramphenicol kanamycin	*E. coli* MG1655 *Δara*	Generous gift from F. Dionisio and J. Alves Gama (Gama et al, 2020 and Prensky et al, 2021)[7,8]
R64drd	I1	120826	5.81	0.51	streptomycin **tetracycline**	*E. coli* DH5*a*	Generous gift from F. Dionisio and J. Alves Gama (Gama et al, 2020 and Prensky et al, 2021)[7,8]
R6kdrd	X	39872	6.51	0.26	**carbenicillin**	*E. coli* DH5*a*	Generous gift from D. Mazel (Baharoglu et al,

							2010 and Prensky, H., et al 2021)[6,8]
R702	P	76639	5.93	0.53	kanamycin<br>spectinomycin<br>streptomycin<br>**tetracycline**	*E. coli* MG1655 *Δara*	Generous gift from F. Dionisio and J. Alves Gama (Gama et al, 2020)[7]
RIP113	N	42161	5.19	0.21	**tetracycline**	*E. coli* DH5*a*	Generous gift from D. Mazel (Baharoglu et al, 2010)[6]
RIP71a	F	91490	2.61	0.41	carbenicillin<br>chloramphenicol<br>spectinomycin<br>**streptomycin**<br>tetracycline	*E. coli K12*	Generous gift from E. Top (Król et al, 2013)[9]
RN3	N	57533	4.60	0.99	spectinomycin<br>streptomycin<br>**tetracycline**	*E. coli* MG1655 *Δara*	Generous gift from F. Dionisio and J. Alves Gama (Gama et al, 2020)[7]
RP4	P	60096	6.63	0.20	amoxicillin/<br>clavulanate<br>carbenicillin<br>kanamycin<br>**tetracycline**	*E. coli* DA32838	Prensky et al, 2021[8]
Rs-a	W	40060	2.96	0.089	chloramphenicol<br>kanamycin<br>spectinomycin<br>**streptomycin**	*E. coli* DH5*a*	Generous gift from D. Mazel (Baharoglu et al, 2010)[6]
pB10	P	64508	6.14	0.52	streptomycin<br>tetracycline	*E. coli BW255113 ΔacrA*	Generous gift from E. Top (Król et al, 2013)[9]
pCU1	N	39884	5.61	0.34	**carbenicillin**<br>spectinomycin<br>streptomycin	*E. coli* DA32838	Lopatkin, A.J., et al, 2016[10]
pESBL283	I	110137	5.46	0.085	**carbenicillin**<br>spectinomycin<br>streptomycin<br>trimethoprim	*E. coli* DA32838	Lopatkin, A.J., et al (2016)[10]
pKpQILD 2	F	111742	4.42	0.76	amoxicillin/<br>clavulanate<br>**carbenicillin**	*Ecl8*	Generous gift from M. Bruckner (Buckner et al, 2018)[11]
pOX38	F	60270	5.77	0.48	tetracycline	*E. coli MG1655*	Lab stock
pRK100	F	142357	2.31	0.23	carbenicillin<br>**tetracycline**	*E. coli MG1655/pCDF*	Prensky et al, 2021[8]
pIP1100	X	66166	5.01	0.15	amoxicillin/<br>clavulanate<br>carbenicillin<br>gentamicin<br>kanamycin<br>spectinomycin<br>**streptomycin**	*E. coli K12*	Generous gift from E. Top[9]
pIP162	F	87543	2.91	0.20	**carbenicillin**<br>chloramphenicol	*E. coli K12*	Generous gift from E. Top[9]

					spectinomycin<br>streptomycin<br>tetracycline		

[1] Average CFU (colony forming unit/mL) represents the number of *de novo* transconjugants generated by conjugating each plasmid with recipient RB933 (1 hour in M9CA at 25°C)

[2] Drug concentrations are as follows: amoxicillin/clavulanate (60 μg/mL), carbenicillin (100 μg/mL), chloramphenicol (30 μg/mL), gentamicin (50 μg/mL), kanamycin (50 μg/mL), rifampicin (50 μg/mL), spectinomycin (50 μg/mL), streptomycin (50 μg/mL), tetracycline (15 μg/mL), trimethoprim (10 μg/mL). Antibiotics corresponding to calculated average CFU and standard deviation columns are bolded.

C. Colony numbers (n) and number of pooled experiments used for statistics in main Figures:

**Recipient**	**Plasmid**	**drug**	**num_denovo**	**num_adapted**	**num_pooled_exps**
RB933	R100-1	strep	64	125	3
RB933	R100-1	tet	99	117	5
RB933	R16a	carb	37	64	3
RB933	R1drd	carb	37	56	3
RB933	R57b	carb	29	119	4
RB933	R64drd	tet	71	64	3
RB933	R6kdrd	carb	58	205	6
RB933	R702	strep	70	103	3
RB933	R702	tet	48	93	3
RB933	RIP113	tet	139	117	5
RB933	RIP71a	strep	29	95	2
RB933	RN3	tet	110	155	3
RB933	RP4	tet	96	208	7
RB933	Rs-a	strep	29	167	2
RB933	pB10	tet	193	190	6
RB933	pCU1	carb	58	145	4
RB933	pESBL283	carb	92	52	2
RB933	pESBL283	strep	26	74	2
RB933	pKpQILD2	carb	42	76	3
RB933	pOX38	tet	51	164	6
RB933	pRK100	tet	35	269	4
RB933	pip1100	strep	33	67	2
RB933	pip162	carb	140	26	2
KPN0029	RIP113	tet	31	60	2
KPN0047	RIP113	tet	30	38	2
KPN0049	RIP113	tet	17	29	2
KPN0054	RIP113	tet	30	29	2
KPN0064	RIP113	tet	9	12	2
KPN0064	RP4	tet	12	33	2
KPN0087	RIP113	tet	106	80	2
KPN0106	RIP113	tet	53	40	2
KPN0112	RIP113	tet	23	112	2
KPN0127	RIP113	tet	71	93	2
KPN0159	RIP113	tet	55	78	2
KPN0159	RP4	tet	27	56	2
KPN0165	RIP113	tet	20	46	2
KPN0169	RIP113	tet	47	65	2
KPN0169	RP4	tet	25	65	2
KPN0193	RIP113	tet	22	62	2

KPN0200	RIP113	tet	21	31	2
KPN0200	RP4	tet	22	48	2
KPN0201	RIP113	tet	48	36	2
KPN0201	RP4	tet	36	36	2
KPN0212	RIP113	tet	25	58	2
KPN0224	RIP113	tet	30	57	2
KPN0224	RP4	tet	24	67	2
KPN0229	RIP113	tet	71	118	2
KPN0229	RP4	tet	53	156	2
KPN0243	RIP113	tet	26	32	2
KPN0267	RIP113	tet	13	14	2
KPN0284	RIP113	tet	14	68	2
KPN0306	RIP113	tet	8	57	2
KPN0337	RIP113	tet	29	45	2

Supplementary Table 2: Analysis of the tet operon and its relationship to acquisition costs

A. Distance matrix for multisequence alignment. Distance matrix based on multi-sequence alignment for all tet operons from the 9 tetracycline plasmids.

	RP4	pRK100	pB10	R702	RN3	R100-1	R64drd	pOX38	RIP113
RP4		99.64	99.64	99.59	93.12	43.62	32.27	32.32	35.16
pRK100	99.64		100	99.95	93.38	50.64	32.42	32.47	35.35
pB10	99.64	100		99.95	93.38	50.64	32.42	32.47	35.35
R702	99.59	99.95	99.95		93.33	50.59	32.47	32.52	35.3
RN3	93.12	93.38	93.38	93.33		49.85	32.08	32.13	34.07
R100-1	43.62	50.64	50.64	50.59	49.85		31.95	32	30.37
R64drd	32.27	32.42	32.42	32.47	32.08	31.95		99.9	50.82
pOX38	32.32	32.47	32.47	32.52	32.13	32	99.9		50.92
RIP113	35.16	35.35	35.35	35.3	34.07	30.37	50.82	50.92	

B. Correlations between acquisition cost and distance between TetA and *oriT/C*. Distance between *oriT(C)* end nucleotide and *tetA* start was calculated, and correlation based on linear regression is reported (bottom row).

**Plasmid**	**AC**	**Distance_oriT**	**Distance_oriC**
R100-1	1.0222	84917	90539
R64drd	1.1898	65315	122336
pOX38	1.2123	112398	75487
RIP113	1.3204	51732	57469
RN3	1.4717	25267	68492
R702	1.4744	42936	9332
RP4	1.4878	22772	60852
pB10	1.5388	80822	69130
pRK100	1.7857	91557	137643
Regression correlation p-value from two-sided t-tests:		**p = 0.57**	**p = 0.82**

Supplementary Table 3: Summary of experimental conditions used in our study

Plasmid	Selection (recipient/donor)	Recipient RB933	Recipient BW25113-rif	Recipient *KPN*
R100-1	rifampicin/streptomycin	Fig. 2d, 4a; Supplementary Fig. 6a		
R100-1	rifampicin/tetracycline	Fig. 2a-d, 3a-b, 4a-b; Supplementary Fig. 2, 3, 4, 6a		
R100-1	kanamycin/tetracycline	Supplementary Fig. 4		
R16a	rifampicin/carbenicillin	Fig. 2d, 4a; Supplementary Fig. 6a		
R1drd	rifampicin/carbenicillin	Fig. 2d, 4a; Supplementary Fig. 6a		
R57b	rifampicin/carbenicillin	Fig. 2d, 4a; Supplementary Fig. 6a		
R64drd	rifampicin/tetracycline	Fig. 2a-d, 3a-b, 4a Supplementary Fig. 2, 3, 6a	Fig. 4f; Supplementary Fig. 7, 8	
R6kdrd	rifampicin/carbenicillin	Fig. 2d, 4a; Supplementary Fig. 6a		
R702	rifampicin/streptomycin	Fig. 2d, 4a; Supplementary Fig. 6a		
R702	rifampicin/tetracycline	Fig. 2a-d, 3a-b, 4a Supplementary Fig. 2, 3, 6a		
RIP113	rifampicin/tetracycline	Fig. 2a-d, 3a-b, 4a; Supplementary Fig. 2, 3, 4, 6a		Fig. 2e; Supplementary Fig. 5
RIP113	kanamycin /tetracycline	Supplementary Fig. 4		
RIP71a	rifampicin/streptomycin	Fig. 2d, 4a; Supplementary Fig. 6a		
RN3	rifampicin/tetracycline	Fig. 2a-d, 3a-b, 4a; Supplementary Fig. 2, 3, 6a		
RP4	rifampicin/tetracycline	Fig. 1c-e, 2a-d, 3a-b, 4a, 4c; Supplementary Fig. 2, 3, 6a	Fig. 4f; Supplementary Fig. 7, 8	Fig. 2e; Supplementary Fig. 5
Rs-a	rifampicin/streptomycin	Fig. 2d, 4a; Supplementary Fig. 6a		
pB10	rifampicin/tetracycline	Fig. 2a-d, 3a-b, 4a; Supplementary Fig. 2, 3, 6a	Fig. 4f; Supplementary Fig. 6b, 7, 8	
pCU1	rifampicin/carbenicillin	Fig. 2d, 4a; Supplementary Fig. 6a		
pESBL283	rifampicin/carbenicillin	Fig. 2d, 4a; Supplementary Fig. 6a		
pESBL283	rifampicin/streptomycin	Fig. 2d, 4a; Supplementary Fig. 6a		
pKpQILD2	rifampicin/carbenicillin	Fig. 2d, 4a; Supplementary Fig. 6a		
pOX38	rifampicin/tetracycline	Fig. 2a-d, 3a-b, 4a; Supplementary Fig. 2, 3, 6a		
pRK100	rifampicin/tetracycline	Fig. 2a-d, 3a-b, 4a; Supplementary Fig. 2, 3, 6a		
pIP1100	rifampicin/streptomycin	Fig. 2d, 4a; Supplementary Fig. 6a		
pIP162	rifampicin/carbenicillin	Fig. 2d, 4a; Supplementary Fig. 6a		

Supplementary Table 4: ODE model parameters

A. Adapted and *De novo* ODE parameter values

**Parameter**	**Adapted value**	***De novo* value**	**Units**	**Description**
μ_m	RP4: 0.5<br>R100-1: 0.4<br>pB10: 0.5<br>R64drd: 0.225	RP4: 0.3-0.5<br>R100-1: 0.4<br>pB10: 0.5<br>R64drd: 0.225	$Hours^{-1}$	Average growth rate
λ	RP4: 8<br>R100-1: 8<br>pB10: 8<br>R64drd: 8	RP4: 10<br>R100-1: 8<br>pB10: 16<br>R64drd: 8	Hours	Average lag time
m	RP4: 0<br>R100-1: 0<br>pB10: 0<br>R64drd: 0	RP4: 0.5<br>R100-1: 0<br>pB10: 0.7<br>R64drd: 0	$Hours^{-2}$	Slope of growth rate-lag time tradeoff

B. General ODE simulation parameters and state variables

**Parameter**	**Value**	**Units**	**Description**
N_m	5e8	Cells/mL	Carrying capacity
Variance	0.002-0.1		Standard deviation for lognormal distributions
N_0	3e1-3e5	Cells/mL	Initial number of *de novo* or adapted clones
P_0	1e3*N_0	Cells/mL	Initial number of background parents in *de novo* mixture
μ_{j+1}	0	$Hours^{-1}$	Growth rate of parents
λ_{j+1}	0	Hours	Lag time of parents
Initial ratio	RP4:pB10 – 1:1<br>RP4:R64drd – 1:20		Ratio of initial mixture
Iterations	50		
t	0:48	Hours	Duration of simulations

References

1. Zwietering, M. H., Jongenburger, I., Rombouts, F. M. & van 't Riet, K. Modeling of the Bacterial Growth Curve. *Appl Environ Microbiol* **56**, 1875–1881 (1990).
2. McKellar, R. C. & Hawke, A. Assessment of distributions for fitting lag times of individual cells in bacterial populations. *International Journal of Food Microbiology* **106**, 169–175 (2006).
3. Huang, L. Simulation and evaluation of different statistical functions for describing lag time distributions of a bacterial growth curve. *Microbial Risk Analysis* **1**, 47–55 (2016).
4. Leónidas Cardoso, L., Durão, P., Amicone, M. & Gordo, I. Dysbiosis individualizes the fitness effect of antibiotic resistance in the mammalian gut. *Nat Ecol Evol* **4**, 1268–1278 (2020).
5. Gomez-Simmonds, A. *et al.* Population Structure of Klebsiella pneumoniae Causing Bloodstream Infections at a New York City Tertiary Care Hospital: Diversification of Multidrug-Resistant Isolates. *J Clin Microbiol* **53**, 2060–2067 (2015).
6. Baharoglu, Z., Bikard, D. & Mazel, D. Conjugative DNA Transfer Induces the Bacterial SOS Response and Promotes Antibiotic Resistance Development through Integron Activation. *PLoS Genet* **6**, e1001165 (2010).
7. Gama, J. A., Zilhão, R. & Dionisio, F. Plasmid Interactions Can Improve Plasmid Persistence in Bacterial Populations. *Frontiers in Microbiology* **11**, (2020).
8. Prensky, H., Gomez-Simmonds, A., Uhlemann, A. & Lopatkin, A. J. Conjugation dynamics depend on both the plasmid acquisition cost and the fitness cost. *Molecular Systems Biology* **17**, (2021).
9. Król, J. E. *et al.* Invasion of E. coli biofilms by antibiotic resistance plasmids. *Plasmid* **70**, 110–119 (2013).
10. Lopatkin, A. J. *et al.* Antibiotics as a selective driver for conjugation dynamics. *Nat Microbiol* **1**, 16044 (2016).

11. Buckner, M. M. C. *et al.* Clinically Relevant Plasmid-Host Interactions Indicate that Transcriptional and Not Genomic Modifications Ameliorate Fitness Costs of Klebsiella pneumoniae Carbapenemase-Carrying Plasmids. *mBio* **9**, e02303-17 (2018).
