# Supplementary material for: Tradeoff between lag time and growth rate drives the plasmid acquisition cost
Source: Nat Commun. 2023 Apr 24;14:2343. doi: 10.1038/s41467-023-38022-6 (PMC10126158; doi:10.1038/s41467-023-38022-6)
Supplement: Supplementary file 2 — Reporting Summary [file 41467_2023_38022_MOESM2_ESM.pdf]

nature portfolio

Corresponding author(s): DBPR-22-47301A

Last updated by author(s): Apr 3, 2023

Reporting Summary

Nature Portfolio wishes to improve the reproducibility of the work that we publish. This form provides structure for consistency and transparency in reporting. For further information on Nature Portfolio policies, see our Editorial Policies and the Editorial Policy Checklist.

Statistics

For all statistical analyses, confirm that the following items are present in the figure legend, table legend, main text, or Methods section.

n/a	Confirmed	
☐	☒	The exact sample size (n) for each experimental group/condition, given as a discrete number and unit of measurement
☐	☒	A statement on whether measurements were taken from distinct samples or whether the same sample was measured repeatedly
☐	☒	The statistical test(s) used AND whether they are one- or two-sided<br>*Only common tests should be described solely by name; describe more complex techniques in the Methods section.*
☐	☒	A description of all covariates tested
☐	☒	A description of any assumptions or corrections, such as tests of normality and adjustment for multiple comparisons
☐	☒	A full description of the statistical parameters including central tendency (e.g. means) or other basic estimates (e.g. regression coefficient) AND variation (e.g. standard deviation) or associated estimates of uncertainty (e.g. confidence intervals)
☐	☒	For null hypothesis testing, the test statistic (e.g. F, t, r) with confidence intervals, effect sizes, degrees of freedom and P value noted<br>*Give P values as exact values whenever suitable.*
☒	☐	For Bayesian analysis, information on the choice of priors and Markov chain Monte Carlo settings
☒	☐	For hierarchical and complex designs, identification of the appropriate level for tests and full reporting of outcomes
☐	☒	Estimates of effect sizes (e.g. Cohen's d, Pearson's r), indicating how they were calculated

Our web collection on statistics for biologists contains articles on many of the points above.

Software and code

Policy information about availability of computer code

Data collection	All data was collected using freely available tools and softwares (i.e., linux scanimage command, CLUSTALW 2.1, iControl Tecan software V3.6.01). All tetracycline analysis code is archived on Zenodo at the following DOI: 10.5281/zenodo.7753403 (https://zenodo.org/badge/latestdoi/606017397). It is also available on the lab's Github: https://github.com/ajlopatkin/acquisition_cost_tet_analysis. All image analysis code is also archived on Zenodo at the following DOI: 10.5281/zenodo.7753412 (https://zenodo.org/badge/latestdoi/557526918), and on the lab's Github: https://github.com/ajlopatkin/acquisition_cost_image_analysis
Data analysis	All image analysis was conducted using custom MATLAB software that is publicly available on the lab's github, and is archived at the zenodo link provided above.

For manuscripts utilizing custom algorithms or software that are central to the research but not yet described in published literature, software must be made available to editors and reviewers. We strongly encourage code deposition in a community repository (e.g. GitHub). See the Nature Portfolio guidelines for submitting code & software for further information.

Data

Policy information about availability of data

All manuscripts must include a data availability statement. This statement should provide the following information, where applicable:

- Accession codes, unique identifiers, or web links for publicly available datasets
- A description of any restrictions on data availability
- For clinical datasets or third party data, please ensure that the statement adheres to our policy

All raw data is submitted in the supplement and provided as source data files. The GenBank database was used to store plasmid sequences, and associated accession IDs are provided.
Data Availability Statement: All data generated in this study are provided in the Supplementary Information and/or as a Source Data file. The plasmid assemblies used in this study are available under the following Genbank accession IDs: [R100-1 (OQ683449), R64drd (OQ683450), R702 (OQ683451), RIP113 (OQ683452), RN3 (OQ683453), and pOX38 (OQ683454)], and archived on the Zenodo database at the following DOI: 10.5281/zenodo.7753403.

Human research participants

Policy information about studies involving human research participants and Sex and Gender in Research.

Reporting on sex and gender	NA
Population characteristics	NA
Recruitment	NA
Ethics oversight	NA

Note that full information on the approval of the study protocol must also be provided in the manuscript.

Field-specific reporting

Please select the one below that is the best fit for your research. If you are not sure, read the appropriate sections before making your selection.

☒ Life sciences ☐ Behavioural & social sciences ☐ Ecological, evolutionary & environmental sciences

For a reference copy of the document with all sections, see nature.com/documents/nr-reporting-summary-flat.pdf

Life sciences study design

All studies must disclose on these points even when the disclosure is negative.

Sample size	All main experiments were conducted in at least three independent biological replicates according to field standards. Sample sizes were chosen for single-colony work by calibrating cell densities to: (1) maximize colony counts while ensuring colonies remain at single-cell resolution for accurate detection by computational software and (2) to avoid density-dependent effects. Colonies are pooled across independent replicates and used for statistics. For some datasets that were particularly large, only two independent replicates that still retained more than 30 colonies each were used. In all cases, replica plots were used to ensure qualitative trends were maintained, which they were. Since our study investigates heterogeneity at the single colony level, individual colonies are considered unique replicates and therefore our results are biologically conclusive.
Data exclusions	All data exclusions are clearly stated in the methods. Briefly, data was excluded if LB media negative controls showed growth, indicating contamination. Other reagents prone to contamination (milliq water, minimal growth media) were also routinely assessed for contamination; data was excluded if contamination was observed. Additionally, colonies were excluded if they could not be confidently separated and analyzed as single colonies, a key requirement in our study. Finally, colonies were excluded if they did not reach necessary thresholds, such as growing to the necessary density threshold.
Replication	All primary experiments were conducted in triplicates (i.e., all tetracycline data and any/all competition experiments); most experiments were conducted with two independent experimentalists. All attempts at replication were successful. For generality experiments, some conditions were conducted in duplicates due to sufficient colony numbers, which make up our true sample size. These numbers are all clearly reported in Supplementary Table 1c.
Randomization	Data was not randomized; multiple replicates of all plasmids in different hands with blinded analysis was sufficient for rigorous and reproducible data. Covariates were controlled for by: conducting multiple biological replicates on different days or with different experimentalists, starting single-colony work with unique single colonies and using multiple-drug conditions, species, and recipient strains to generalize conclusions to present rigorous and reproducible data.
Blinding	All analysis that required manual intervention (i.e., excluding colonies) was blinded with respect to the experimental condition (i.e., the

analyzer did not know the condition they were evaluating), as described in the methods.

Reporting for specific materials, systems and methods

We require information from authors about some types of materials, experimental systems and methods used in many studies. Here, indicate whether each material, system or method listed is relevant to your study. If you are not sure if a list item applies to your research, read the appropriate section before selecting a response.

Materials & experimental systems

n/a	Involved in the study
☒	☐ Antibodies
☒	☐ Eukaryotic cell lines
☒	☐ Palaeontology and archaeology
☒	☐ Animals and other organisms
☒	☐ Clinical data
☒	☐ Dual use research of concern

Methods

n/a	Involved in the study
☒	☐ ChIP-seq
☒	☐ Flow cytometry
☒	☐ MRI-based neuroimaging
